# Supplementary material for: Tead1a Initiates Transcriptional Priming Through the TEAD1a/YAP‐Notch1‐Spi1/Cebpα Axis to Promote Neutrophil Fate
Source: Adv Sci (Weinh). 2025 Aug 28;12(41):e05441. doi: 10.1002/advs.202505441 (PMC12591127; doi:10.1002/advs.202505441)
Supplement: Supplementary file 4 — Supplementary Dataset [file ADVS-12-e05441-s004.zip › 3-1.zip!/3-1/02-1.cluster/cluster2/Enrich/ego_BP.pdf]

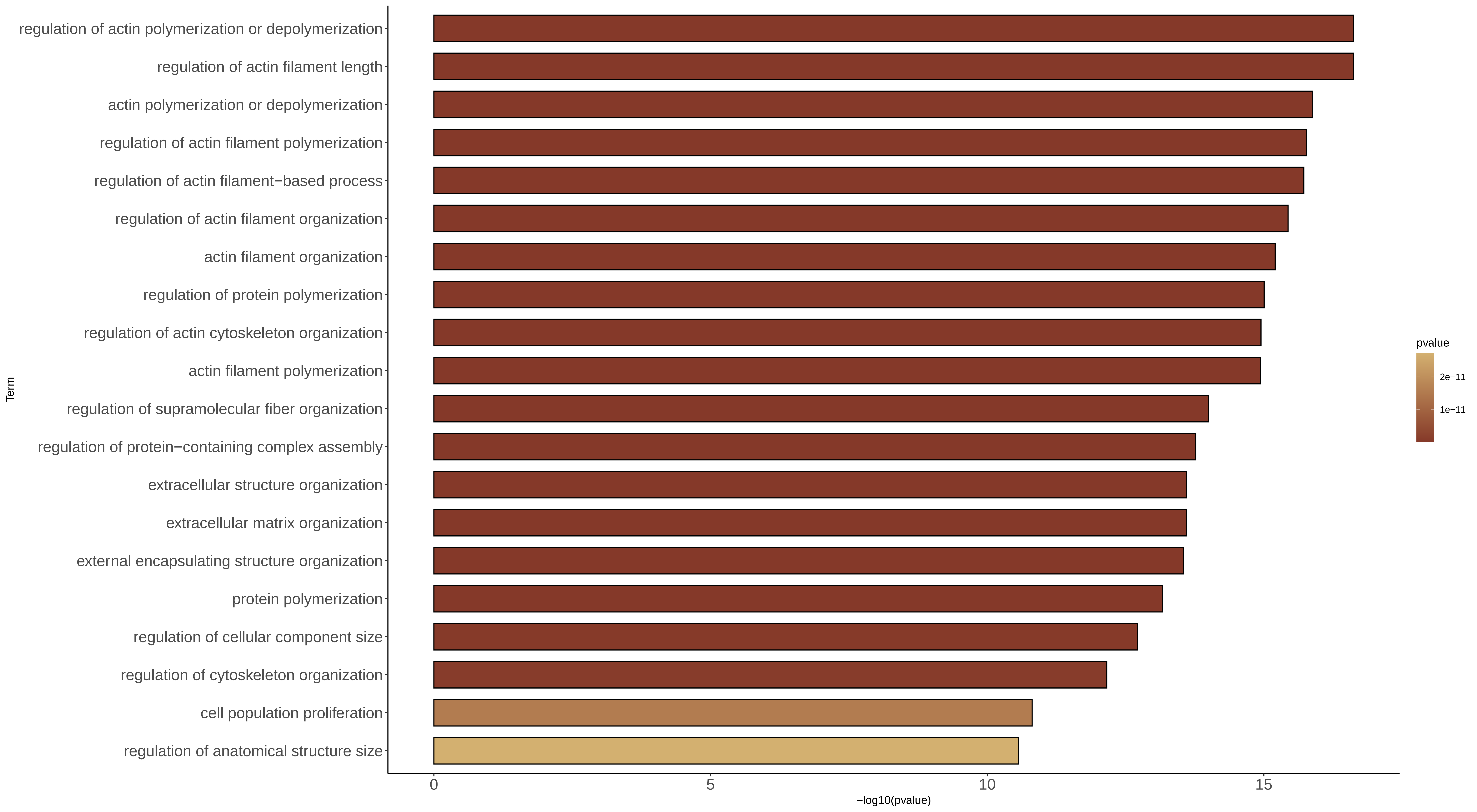
regulation of actin polymerization or depolymerization
regulation of actin filament length
actin polymerization or depolymerization
regulation of actin filament polymerization
regulation of actin filament−based process
regulation of actin filament organization
actin filament organization
regulation of protein polymerization
regulation of actin cytoskeleton organization
actin filament polymerization
regulation of supramolecular fiber organization
regulation of protein−containing complex assembly
extracellular structure organization
extracellular matrix organization
external encapsulating structure organization
protein polymerization
regulation of cellular component size
regulation of cytoskeleton organization
cell population proliferation
regulation of anatomical structure size
Term
0
5
10
15
−log10(pvalue)
pvalue
2e−11
1e−11
